# Molecular Interaction of a New Antibacterial Polymer with a Supported Lipid Bilayer Measured by an *in situ* Label-Free Optical Technique

**DOI:** 10.3390/ijms14059722

**Published:** 2013-05-06

**Authors:** Robert Horvath, Balázs Kobzi, Helmut Keul, Martin Moeller, Éva Kiss

**Affiliations:** 1 MTA TTK MFA Institute for Technical Physics and Materials Science, Research Centre for Natural Sciences, Budapest, Konkoly Thege u. 29-33 H-1121, Hungary; E-Mail: horvathr@mfa.kfki.hu; 2 Laboratory of Interfaces and Nanostructures, Institute of Chemistry, Eötvös Loránd University, P.O. Box 32, Budapest 112 H-1518, Hungary; E-Mails: kobzibalazs@gmail.com (B.K.); kisseva@chem.elte.hu (É.K.); 3 DWI an der RWTH Aachen e.V. and Institute of Technical and Macromolecular Chemistry, RWTH Aachen, Forckenbeckstr. 50, Aachen D-52056, Germany; E-Mail: moeller@dwi.rwth-aachen.de

**Keywords:** antibacterial polymer, supported lipid bilayer, optical waveguide spectroscopy, optical anisotropy, label-free measurement

## Abstract

The interaction of the antibacterial polymer–branched poly(ethylene imine) substituted with quaternary ammonium groups, PEO and alkyl chains, PEI_25_QI_5_J_5_A8_15_–with a solid supported lipid bilayer was investigated using surface sensitive optical waveguide spectroscopy. The analysis of the optogeometrical parameters was extended developing a new composite layer model in which the structural and optical anisotropy of the molecular layers was taken into consideration. Following *in situ* the change of optical birefringence we were able to determine the composition of the lipid/polymer surface layer as well as the displacement of lipid bilayer by the antibacterial polymer without using additional labeling. Comparative assessment of the data of layer thickness and optical anisotropy helps to reveal the molecular mechanism of antibacterial effect of the polymer investigated.

## 1. Introduction

Cell walls or membranes control the chemical communication between otherwise isolated compartments in the living organisms. In spite of their complex structure, their permeability is highly governed by the lipid bilayer membrane. The most characteristic feature of the lipid bilayer is the organization of lipid molecules into two leaflets positioned face-to-face with ordered alignment of the alkyl chains. That special architecture of the closely packed assembly is responsible for the barrier function. The antibacterial agents act by destroying this barrier function of the bacterial cell wall leading to disintegration of bacterial cell membrane [1–6].

Phospholipid assemblies as liposomes or planar lipid bilayers formed on a solid support (supported lipid bilayer, SLB) are convenient model systems to examine the influence of bioactive components or nanoparticles [7–12] on the structural order of lipid bilayers resulting in its permeabilization or destabilization. One of the well described mechanisms is the barrel-stave model, while the other is the carpet (detergent-like) mode of action [13–16]. Both mechanisms mainly refer to natural and synthetic antibacterial peptides. The mechanism by which an antimicrobial polymer penetrates and disrupts the phospholipid bilayer is not completely understood. The investigation of polymeric type antibacterial agents and their interaction with planar lipid bilayers is sporadic [5]. Characterizing such interactions however, is of primary importance since recent studies demonstrated the high efficiency and exceptional advantages of such materials [17–21]. Providing non-elutable coverage on various surfaces such as of medical devices or food packaging materials these antibacterial polymers are ideal substances for bactericid, fungicide or virucid surface treatment [22,23]. Most water soluble antimicrobial polymers are linear [24–26]. Newly designed and synthesized cationic amphipathic polyelectrolytes based on branched poly(ethylene imine) (PEI), provide a means to control the molecular shape and surface interaction. The polymers were prepared reacting the primary amino groups of PEI with ethylene carbonate functional couplers bearing cationic groups, poly(ethylene oxide), and alkyl chains of different lengths. The functionalized PEIs present considerable surface activity and antibacterial properties [27,28]. Detailed knowledge of their action will contribute to the rational design and functional improvement.

Different aspects of interaction of bioactive material with supported lipid bilayer were studied by various techniques such as sum frequency generation spectroscopy [14,29], atomic force microscopy [30,31], cyclic voltammetry [32], nuclear magnetic resonance [33,34], quarz crystal microbalance [35,36], surface plasmon resonance [37], fluorescence spectroscopy [38] or Fourier-transformed infrared spectroscopy [39] as well as optical waveguide methods. The waveguide spectroscopy techniques as dual polarization interferometry (DPI) [40,41], coupled plasmon waveguide resonance (CPWR) [42,43], and optical waveguide lightmode spectroscopy (OWLS) [44–51] are especially valuable among those because of the possibility to deduce the presence and integrity of the bilayer, which is a crucial functional property [10]. Further advantages of these techniques are the real time and label-free measurement of the optical properties of nanometer scale films in aqueous solutions [52–57].

In the present work branched PEI functionalized with cationic groups, as well as hydrophilic and alkyl chains, PEI_25_QI_5_J_5_A8_15_ [20], was used to determine its interaction and exchange with solid supported lipid, POPC, bilayers applying the OWLS technique. During the measurements the lipid bilayer was formed on the surface of the optical waveguide chip *in situ* in the OWLS instrument by lipid vesicle rupture [44,58,59]. After obtaining a compact bilayer, the antibacterial polymer was introduced continuously. The fluid flow provides a constant concentration profile during the measurement thereby the condition resembles to the bacterial contact with a permanent antibacterial coating.

## 2. Results and Discussion

Effect of antibacterial polymer on the supported lipid bilayer was studied by the OWLS technique. During the measurement the formation of lipid bilayer as well as its interaction with the injected antibacterial polymer was followed by recording the change in the effective refractive indices of the modes of the waveguide [60–62].

These data were used to calculate the optical thickness of the adlayer, *Q =* (*ñ_A_* − *n_C_* )*d̃_A_*, where *ñ_A_* and *n_C_* are the refractive indices of the surface layer (adlayer) and the solution (cover medium), respectively while *d̃_A_* is the thickness of the surface layer obtained using the homogeneous and isotropic layer model [63]. Note, *Q* is an optogeometrical parameter directly proportional to the deposited surface mass density [49,63,64].

**Figure 1:**
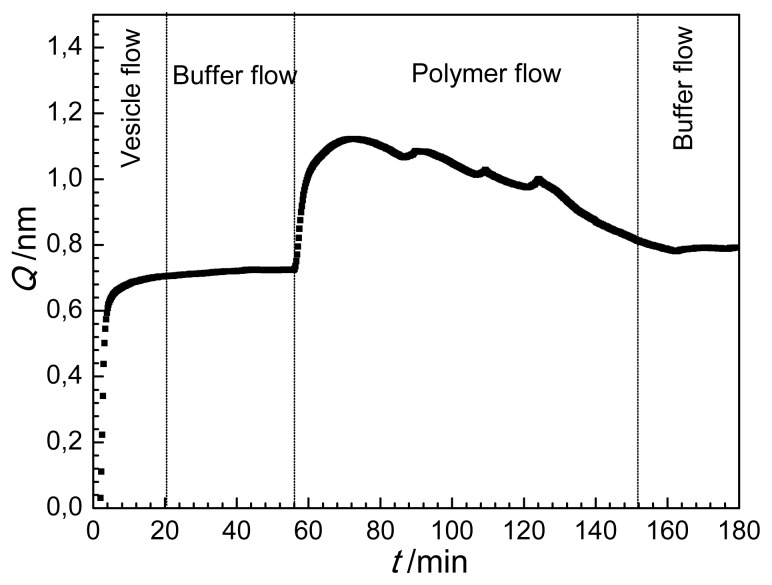
Optical thickness (*Q*) as a function of time (*t*) on the waveguide surface in the course of lipid vesicle/antimicrobial polymer deposition.

After recording a stable baseline (no adlayer on the surface, *Q =* 0 ) the lipid vesicle suspension was injected onto the surface in order to create a supported lipid bilayer by vesicle rupture and spreading (see Figure 1). This leads to a rapid increase in the optical thickness reaching a saturation value after about 12 min. Then pure buffer was flowed into the cuvette (washing). During the buffer flow the supported lipid bilayer was stable, showing no significant change in the optical thickness. Injecting the solution of antibacterial polymer into the cuvette a fast increase of the layer thickness was observed. After reaching a maximum value (at about 70 min) the optical thickness, interestingly, started to decrease while still injecting the polymer solution into the cuvette. A *Q* value close to the optical thickness of the pure lipid bilayer was reached at about 150 min. The continuously decreasing tendency of the optical thickness has stopped when pure buffer was flowed into the cuvette and *Q* seemed to be well stabilized (see Figure 1).

The change in optical thickness represents an overall signal characterizing the surface layer during bilayer formation and lipid-polymer interaction, but does not reveal the fine details of the surface processes. Several interesting questions arise especially looking at part of the curve in Figure 1 following the injection of the antibacterial polymer, where both the lipid and polymer are present at the surface. For instance, it would be important to know how the exact composition of the surface layer changes. A further question might be whether the lipid bilayer is displaced by the antibacterial polymer or remains intact after desorption of the firstly adsorbed polymer. These questions, which cannot be answered by investigating the variation in optical thickness only, are in the focus of the present study.

### 2.1. Isotropic Adlayer Model

**Figure 2:**
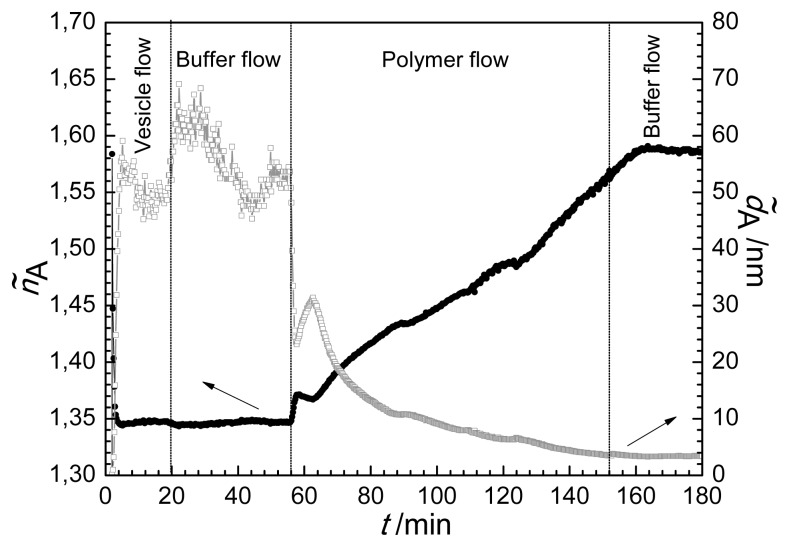
Refractive index (ñ*_A_*) and layer thickness (*d̃_A_*) during lipid vesicle/antimicrobial polymer insertion as a function of time (*t*) using the homogeneous and isotropic layer model (arrows indicate the axis of plotted data).

In order to obtain a deeper insight into the structure of the dynamically changing surface film the thickness (*d̃_A_*) and refractive index (ñ*_A_*) obtained from the homogeneous and isotropic optical adlayer [61,63] were calculated. These data are plotted in Figure 2. It seems that incorrect values were obtained for both layer thickness and refractive index in several intervals of the process. First, the layer thickness of the pure bilayer is strongly overestimated, having values around 50–60 nm in contrast to the real thickness of about 5 nm determined by neutron scattering [10,40]. Moreover, the refractive index close to 1.6, obtained for the film following polymer injection, seems also to be too high [10]. From that we can conclude that the homogeneous and isotropic adlayer model failed to characterize the film.

It should be emphasized that the unrealistic values obtained are in close connection with the anisotropic nature of the film [10,46,63]. Films with structural ordering are usually optically anisotropic [65]. Theoretical calculations showed that the optical birefringence in a layer results in unrealistic thickness and refractive index values when the film is modeled as isotropic [63]. Overestimated quasi-isotropic thickness linked to underestimated quasi-isotropic refractive index (at 4–56 min in Figure 2) originates from positive birefringence (*n*_e_ > *n*_o_), when refractive index perpendicular to the substratum (*n*_e_) is larger than the index parallel to the substratum (*n*_o_). This result on the positive birefringence of the lipid bilayer is in fine agreement with earlier studies of lipid membranes performed with DPI, CPWR techniques [10,43,49]. On the other hand, the overestimated refractive index value is a clear sign of negative birefringence (*n*_e_ < *n*_o_) in the surface layer. The above findings already suggest that, remarkably, the optical anisotropy of the surface layer changes its sign as a result of the interaction of the lipid bilayer with the antibacterial polymer (see Figure 2). It should be noted that the above errors in quasi-isotropic thickness and refractive index compensate each other and hence the determined optical thickness value [*Q =* (*ñ_A_* − *n_C_*)*d̃_A_*] is quite accurate [63].

### 2.2. Anisotropic Adlayer Model

**Figure 3:**
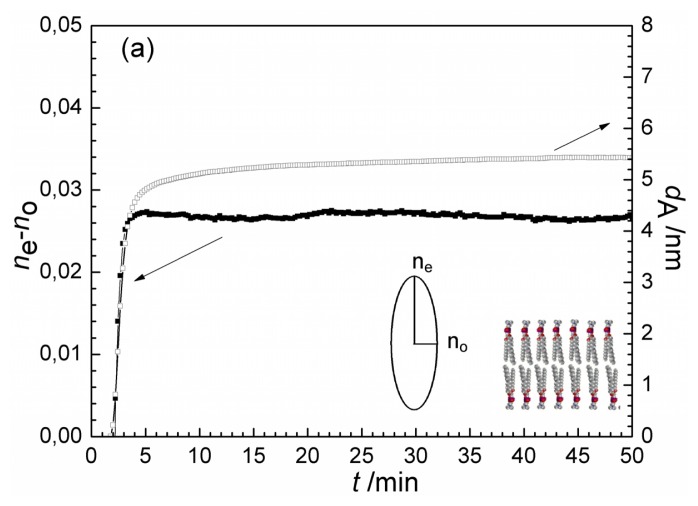

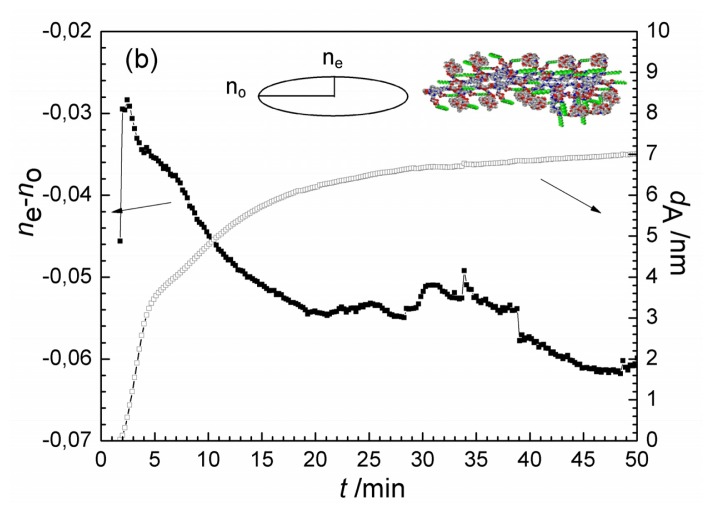
Birefringence (*n*_e_ − *n*_o_) and layer thickness, *d*_A_ obtained during lipid bilayer formation (**a**) and adsorption of the antibacterial polymer; (**b**) using the anisotropic optical adlayer model (arrows indicate the axis of plotted data). The refractive index ellipsoids and the structure of the films are also shown.

To understand deeper the interaction of the antibacterial polymer with the lipid bilayer the optical data determined in the course of lipid/polymer deposition experiment were analyzed by adapting an anisotropic optical layer model [46,63,66]. The anisotropy of the surface deposited layer is defined as the refractive index value perpendicular to the surface (extraordinary, *n*_e_) minus the refractive index experienced parallel to the surface (ordinary, *n*_o_), *i.e.*, *n*_e_ − *n*_o_. The deposited film was therefore characterized by two refractive index values, and by its thickness, *d*_A_ in this approach. During the anisotropic calculations, the averaged refractive index of the layer $\sqrt{(2n_{o}^{2}+n_{e}^{2})/3}$ was constrained to 1.47 [10,66]. The optogeometrical parameters of the lipid bilayer obtained using the anisotropic layer model are displayed in Figure 3a. *Positive* birefringence was found for the lipid bilayer in accordance with the prediction of the above quasi-isotropic analysis. Its value is 0.026 being in reasonable agreement with DPI measurements of Masaghi *et al.* [10]. The calculated thickness of the compact bilayer is 5.4 nm, similar to value reported previously [10,40].

The anisotropic optogeometrical parameters of the adsorbing antibacterial polymer film were also determined. The results are shown in Figure 3b. Negative birefringence was deduced (−0.06 at saturation) for antibacterial polymer film adsorbed on the bare waveguide surface, with a saturation layer thickness of 7.0 nm. The opposite value of the birefringence (compared to the lipid bilayer) is most probably due to difference in the preferred orientation of the highly polarizable C–C bonds in the layer. In contrast to the lipid chains being perpendicular to the solid surface, the polymer chains possibly are mostly parallel with the surface when they are taking up a flattened, extended surface conformation. A similar behavior was observed for surface adsorbed glycoproteins [66] and for denaturated bovine serum albumin spread on a ZrO_2_ surface [52].

**Figure 4:**
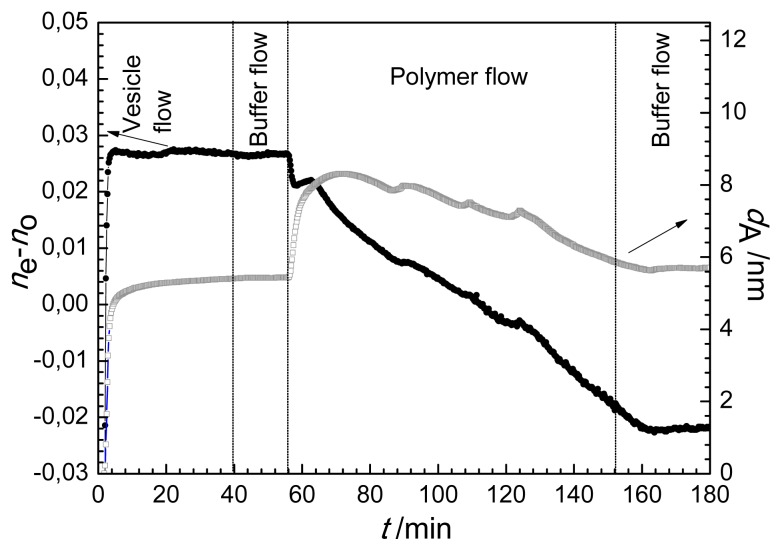
Birefringence (*n*_e_ − *n*_o_) and layer thickness, *d*_A_ during lipid vesicle/antibacterial polymer insertion as a function of time (*t*) using the anisotropic optical layer model (arrows indicate the axis of plotted data).

The calculation based on the anisotropic model was conducted for the experiment when following the formation of lipid bilayer the solution of antibacterial polymer was injected into the OWLS cuvette. The resulted birefringence and layer thickness values are plotted in Figure 4. The layer thickness exhibits similar trend as the optical thickness does (Figure 1), *d*_A_ first increased from 5.2 nm to about 8 nm, and turned to decrease back to a value of 5.8 nm due to the interaction with the antibacterial polymer (see Figure 4). The change of birefringence, however, provides new information on the property and composition of the surface layer. The appearance of antibacterial polymer decreased significantly the anisotropy of the layer, and this effect was continuous during the flow of polymer. At the end of flow of antibacterial polymer (*t =* 152 min) a negative value of the birefringence characterized the surface layer. This clearly shows that the ordered structure of lipid bilayer (with positive birefringence of 0.026) does not hold any more, the conformational structure of the molecules in the surface layer is getting closer to what was obtained for the adsorbed antibacterial polymer (negative birefringence of −0.06).

Considering the layer birefringence (which changed its sign) and thickness data it is suggested that the antibacterial polymer was first adsorbed on top of the compact bilayer (reaching a maximum value after 10 min) later, some parts of the lipid layer were slowly exchanged with the polymer.

### 2.3. Composite Model

We can get a deeper insight into the lipid bilayer-antibacterial polymer molecular interaction if we further analyze the optical data by introducing a composite/exchange model. This allows a mathematical treatment of the composite surface layer composed from purely lipid and purely polymer covered areas. The optogeometrical parameters were deduced from the parameters of the individual components building up the whole layer. During the calculations, we assume that the OWLS method averages the effective refractive index shifts caused by the pure lipid and pure polymer coatings on the surface. This type of averaging of the measuring method was first revealed by Cottier and Horvath using numerical simulations based on the Local Interference Method (LIME) [67]. The straightforward calculations result in the following simple equation for the TE polarization:

(1) $$(n_{o}^{2}-n_{C}^{2})d_{A}=\Theta_{\left(+\right)}(n_{o,(+)}^{2}-n_{C}^{2})d_{\left(+\right)}+\Theta_{\left(-\right)}(n_{o,(-)}^{2}-n_{C}^{2})d_{\left(-\right)}$$

where Θ_(+)_and Θ_(−)_ are the surface coverages of the positively birefringent and negatively birefringent films, respectively. (By definition, the coverage is the area covered by the given component divided by the total sensor area.) *n_o_*_,(+)_ and *d*_(+)_ are the ordinary refractive index and thickness of the positively birefringent component, while *n_o_*_,(−)_ and *d*_(−)_ are the ordinary refractive index and thickness of the negatively birefringent film component. For simplicity, the following approximation was used to calculate the extraordinary refractive index of the composite film *n_e_* from the extraordinary refractive indices of the positively and negatively anisotropic film components; *n_e_*_,(+)_ and *n_e_*_,(−)_, respectively:

(2) $$(n_{e}^{2}-n_{C}^{2})d_{A}=\Theta_{\left(+\right)}(n_{e,(+)}^{2}-n_{C}^{2})d_{\left(+\right)}+\Theta_{\left(-\right)}(n_{e,(-)}^{2}-n_{C}^{2})d_{\left(-\right)}$$

Therefore, by taking the refractive index and thickness values of the pure lipid and polymer films (from Figure 3a,b) and using Equations (1) and (2) the surface coverages of the lipid and polymer covered areas can be calculated. The analysis is therefore based on the different birefringent properties of the two film forming components, Θ_(+)_ relates to the relative area of the sensor surface which is covered by lipid-like material, showing positive birefringence, while Θ_(−)_ represents the area covered by polymer-like molecules showing negative birefringence. It is important to note that Θ_(+)_ + Θ_(−)_ is not necessarily equal to one. If the (fraction of the) chip surface is covered by lipid bilayer and by adsorbed polymer on top as a *second* layer, the sum of the two coverages must be above one. (For example, Θ _(+)_ = 2 with Θ_(− )_ = 0 would mean a second lipid bilayer on top of the first one.)

**Figure 5:**
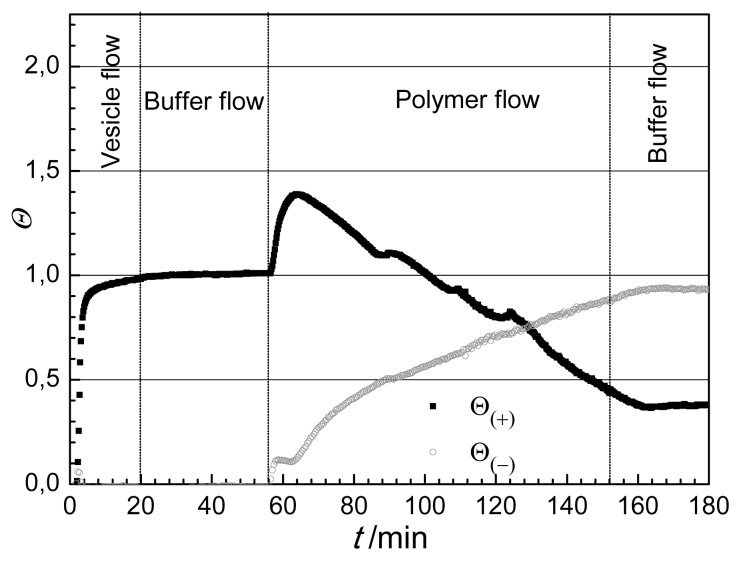
Surface coverages of lipid and polymer on the waveguide calculated from the composite model.

The calculated coverages for the lipid bilayer-polymer deposition process are shown in Figure 5. Looking at the lipid deposition period (until 56 min) the introduced composite model predicts what is expected, Θ_(−)_ = 0 during the whole liposome rupture process, while Θ_(+)_ gradually increases and saturates at 1, indicating the formation of a compact bilayer.

It is seen that injecting the antibacterial polymer at 56 min the coverage by polymer-like molecules Θ_(−)_ starts to increase, while at 64 min saturates and then increases again. The lipid-like coverage, Θ _(+)_, surprisingly, shows a significant increase in the first period of injection of polymer. Afterwards a strong gradual decrease was detected.

**Figure 6:**
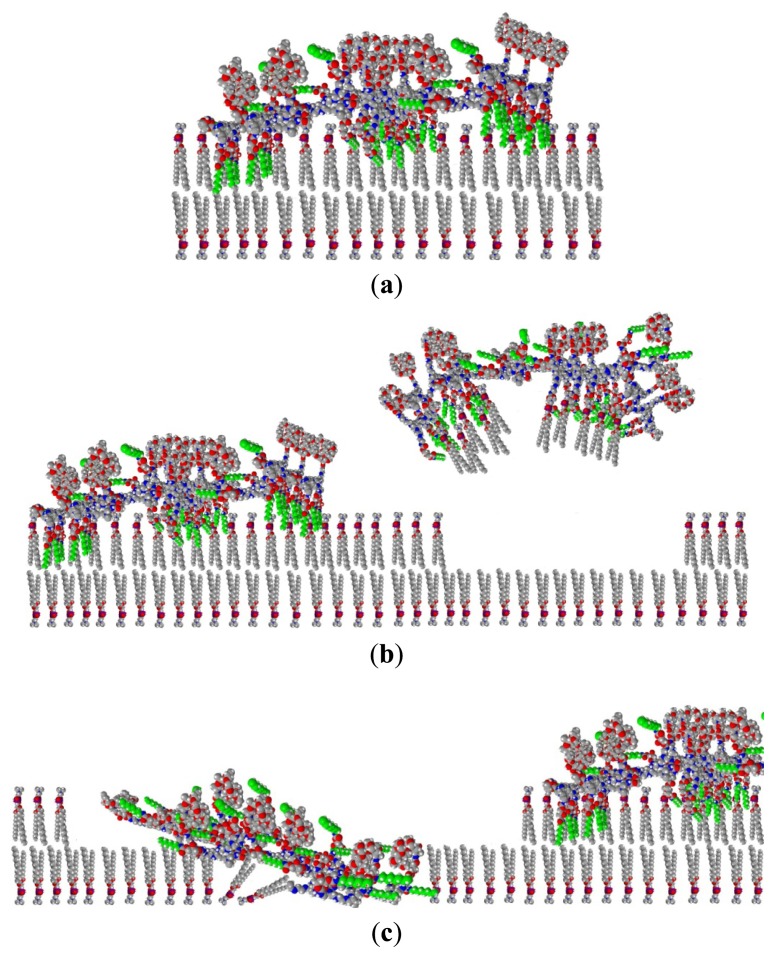
Scheme of the interaction of antibacterial polymer with supported lipid bilayer based on structural information obtained from OWLS measurement using the anisotropic model for data evaluation. The antibacterial polymer is adsorbed on top of the lipid bilayer with penetrating alkyl chains (**a**); antibacterial polymer-lipid complex is leaving the surface (**b**); lipid bilayer is disintegrated and exchanged by the polymer (**c**).

It is interesting to note that the saturation of Θ_(−)_ finishes as Θ_(+)_ starts to decrease (at around 64 min). The above data suggest that the polymer adsorbs on top of the lipid bilayer at the beginning of the interaction process and this adsorption presents a significant contribution not only to the appearance of polymer-like, negatively birefringent component Θ_(−)_, but also to the component with positive birefringent property. That means that the polymer deposited on the top of lipid bilayer must adopt a different conformation than the conformation measured on polymer adsorbed on the bare surface (see conformation scheme in Figure 3b). The perpendicular orientation of alkyl chains to the surface while penetrating into the lipid bilayer is in agreement with this finding (see Figure 6.)

The change in coverages with opposite sign, which is valid in the main part of interaction between 70 and 150 min, corresponds to the partial exchange of the lipid bilayer with the antibacterial polymer. Only after penetrating into the lipid bilayer and forcing the lipid to leave the surface, there is enough space to contact with the bare surface for the polymer and to take up its negatively birefringent extended conformation.

At the end of the interaction and exchange, the coverage of the lipid-like, positively birefringent component is around 40% while the area for the negatively birefringent, polymer like component is 80%. The total coverage is above 100% (see Figure 5). This is clearly suggesting the existence of a multilayered surface area, existing together with the purely lipid covered and purely polymer covered areas. Either some polymers are still covering some parts of the lipids in a conformation with a positive birefringence, or some polymer did not have enough space to take up its fully extended conformation on the bare surface (see Figure 6). Both scenarios would increase the calculated values of the positively anisotropic areas. Therefore, one can conclude that the purely lipid bilayer covered areas are less than 40% at the end of the measurement.

## 3. Experimental Section

### 3.1. Materials

The antibacterial polymer was prepared by functionalization of branched poly(ethylene imine), PEI. The primary amino groups of branched PEI (BASF, Mw: 25,000) were substituted by a quaternary ammonium iodide salt (QI), a poly(ethylene oxide) (PEO, Mw: 1000) derivative (J), and octyl chains (A8) in a well-defined ratio obtaining a polymer with composition of PEI_25_QI_5_J_5_A8_15_. Details of the synthesis and physico-chemical characterization of the polymer were given previously [20].

1-Palmitoyl-2-oleoyl-sn-glycero-3-phosphocholine (POPC) was used to prepare unilamellar lipid vesicles (smaller than 100 nm in diameter), which adsorb and spread on the waveguide’s surface to form the solid supported bilayer [58,59].

### 3.2. OWLS Measurement

Formation of supported lipid bilayer and the study of the effect of antibacterial polymer in contact with that were performed in the flow cell of the OWLS instrument. At first, a grating coupler waveguide sensor chip (OW2400, MicroVacuum, Ltd., Budapest, Hungary) was inserted into the OWLS instrument (BIOS MicroVacuum, Ltd., Budapest, Hungary) capable to record the effective refractive indices of the modes of the waveguide [60–62] with a precision of 10^−6^. The effective refractive indices of the zeroth order transverse electric (TE) and transverse magnetic (TM) polarized modes were saved at every 11.3 s for subsequent data analysis.

To be able to apply the various solutions a PEEK flow-through cuvette with a Kalrez O-ring was placed on top of the waveguide. Pure buffer (saline solution with ionic strength: 0.30, pH = 7.4, NaCl concentration of 0.15 M and with 2 mM CaCl2) without lipid vesicles was injected into the cuvette with a flow rate of 2 μL/s using a peristaltic pump (Ismatec, Wertheim, Germany). After getting a stable baseline lipid vesicle suspension was introduced. Following the formation of lipid bilayer buffer flow was applied again to obtain stable condition. Then the solution of the antibacterial polymer was introduced and its flow was maintained for app. 90 min when a buffer was flowed for about 20 min.

## 4. Conclusions

Summing up, the interaction of the antibacterial PEI_25_QI_5_J_5_A8_15_ polymer with solid supported lipid (POPC) bilayers was investigated using OWLS. As an extension of the analysis of the parameters determined by OWLS technique the optical birefringence of the ordered molecular layer is deduced. That allows for following of the perturbation of the membrane architecture *in situ* by recording the change in optical anisotropy of the surface layer, associated with structural ordering in the film. It was found that the compact lipid bilayer has positive birefringence. The optical birefringence of the antibacterial polyelectrolyte layer adsorbed on the bare surface was also investigated and proved to be negative, opposite to that of the lipid bilayer. Based on this experimental finding a composite optical model was set up in order to deduce the birefringence of the surface layer during the interaction and exchange of the lipid bilayer with the polyelectrolyte. Using the developed model the composition of the lipid-polyelectrolyte surface layer as well as the displacement of lipid bilayer by the antibacterial polyelectrolyte can be estimated without using any additional labeling. This type of information can contribute to the understanding the mechanism of antibacterial effects leading to the disintegration of cell membranes in a more detailed way. Furthermore, the composite model provides an efficient tool in general to analyze molecular interactions of components with different optical anisotropy.
